# Using bioinformatics tools for the discovery of Dengue RNA-dependent RNA polymerase inhibitors

**DOI:** 10.7717/peerj.5068

**Published:** 2018-09-25

**Authors:** Nomagugu B. Nncube, Pritika Ramharack, Mahmoud E.S. Soliman

**Affiliations:** Molecular Bio-computation and Drug Design laboratory, School of Health Sciences, University of KwaZulu-Natal, Durban, KwaZulu-Natal, South Africa

**Keywords:** Dengue drug discovery, RNA-dependent RNA Polymerase, Bioinformatics, *Flavivirus* therapeutics

## Abstract

**Background:**

Dengue fever has rapidly manifested into a serious global health concern. The emergence of various viral serotypes has prompted the urgent need for innovative drug design techniques. Of the viral non-structural enzymes, the NS5 RNA-dependent RNA polymerase has been established as a promising target due to its lack of an enzymatic counterpart in mammalian cells and its conserved structure amongst all serotypes. The onus is now on scientists to probe further into understanding this enzyme and its mechanism of action. The field of bioinformatics has evolved greatly over recent decades, with updated drug design tools now being publically available.

**Methods:**

In this study, bioinformatics tools were used to provide a comprehensive sequence and structural analysis of the two most prominent serotypes of Dengue RNA-dependent RNA polymerase. A list of popular *flavivirus* inhibitors were also chosen to dock to the active site of the enzyme. The best docked compound was then used as a template to generate a pharmacophore model that may assist in the design of target-specific Dengue virus inhibitors.

**Results:**

Comparative sequence alignment exhibited similarity between all three domains of serotype 2 and 3.****Sequence analysis revealed highly conserved regions at residues Meth530, Thr543 Asp597, Glu616, Arg659 and Pro671. Mapping of the active site demonstrated two highly conserved residues: Ser710 and Arg729. Of the active site interacting residues, Ser796 was common amongst all ten docked compounds, indicating its importance in the drug design process. Of the ten docked *flavivirus* inhibitors, NITD-203 showed the best binding affinity to the active site. Further pharmacophore modeling of NITD-203 depicted significant pharmacophoric elements that are necessary for stable binding to the active site.

**Discussion:**

This study utilized publically available bioinformatics tools to provide a comprehensive framework on Dengue RNA-dependent RNA polymerase. Based on docking studies, a pharmacophore model was also designed to unveil the crucial pharmacophoric elements that are required when constructing an efficacious DENV inhibitor. We believe that this study will be a cornerstone in paving the road toward the design of target-specific inhibitors against DENV RdRp.

## Introduction

There are several species under the *flavivirus* genus that continue to cause detrimental effects to infected individuals (King et al., 2007; Holbrook, 2017). One of these species is the Dengue virus (DENV), which is the causative agent of DENV fever (John, 2003; Guzman & Harris, 2015). Upon infection, the mosquito-borne virus may lead to severe flu-like symptoms (Guzman et al., 2010; Ross, 2010).

Studies have shown that approximately 3.9 billion people are prone to DENV infection (Murray, Quam & Wilder-Smith, 2013; World Health Organization (WHO), 2016). The first isolation of the virus was in Japan in 1943. Since then, DENV has disseminated on a global scale, becoming endemic in more than 100 countries. This hyperendemic nature of the virus was likely a result of mosquito vector tansmission, international travel as well as urbanization (Messina et al., 2014). To date, there are currently four DENV serotypes in circulation (Dar et al., 2006; Bharaj et al., 2008; Christenbury et al., 2010). Of these serotypes, serotype 2 and 3 are the most common (Balmaseda et al., 2006; Van Panhuis et al., 2010; Fatima et al., 2011).

Despite the growing number of strains, the RNA-dependent RNA polymerase (RdRp) remains conserved. This RdRp non-structural enzyme also remains specific for viral replication and lacks an enzymatic counterpart in mammalian cells. This allows researchers to utilize this promising target in the design of DENV inhibitors. Despite the constant evolution in this area of research, there still remains no approved antiviral drug or vaccine specific to the RdRp region of DENV (Thomas & Endy, 2011; Lam, 2013; World Health Organization (WHO), 2016). The burden of the virus is further accelerated by the risk of multiple serotype infection. Dengue has an unusual characteristic feature of individual serotype infection leading to homotypic immunity. This could lead to subsequent DENV infections from different serotypes, thus increasing the symptomatic features of the disease. Another detrimental factor in the host response to infection is that both T and B cell mediated retaliation has shown to increase disease pathogenesis in secondary infections (Screaton et al., 2015). It is therefore imperative to source RdRp-specific inhibitors that aim to put an end to the devastating effects of DENV infections.

Information technology has become a critical aspect of the drug discovery process (Hooft, Sander & Vriend, 1997; Huang et al., 2010). Bioinformatics is an emerging scientific domain that is being exploited to replace the old “hand-crafted” synthesis and testing approach (Xu & Hagler, 2002; Chen, 2006; Firdaus Begam & Satheesh Kumar, 2012). The focal point of bioinformatics is to analyze, simulate and manipulate chemical information in order to reduce expenses in the areas of lead compound identification and optimization (Xu & Hagler, 2002; Krasky et al., 2007; Liu et al., 2014). This study utilizes these bio-computational techniques to provide comprehensive informational data that will allow for the identification or design of inhibitors specific to DENV RdRp.

### Methods

Bioinformatics tools were used in this study to analyze the structure of DENV RdRp and map out a potential inhibitor specific to the enzyme.

### Crystal structure acquisition and alignment

The crystal structures of DENV RdRp serotype 2 and 3 were retrieved from the Protein Databank (Berman et al., 2000). Serotype 2 and 3 of DENV are represented by PDB codes 5K5M and 5I3Q, respectively (Lim et al., 2016). The PDB structures were opened simultaneously in Chimera (Pettersen et al., 2004) and superimposed using the Match-maker function. The sequences were then aligned, and regions of similarity were highlighted.

### Sequence and structure analysis

Comparative analysis and structural investigations between the active site regions of the two serotypes were then undertaken using the alignment tool available through Chimera, with default settings applied (Pettersen et al., 2004).

After aligning the two DENV sequences, the conserved regions between the two serotypes were identified. The active site residues were obtained from previous studies (Source et al., 2013; Klema et al., 2016) and validated by identifying the residues interacting with GTP when bound to DENV RdRp (PDB code: 2J7W) using the Chimera visualization software. Important structural features of the RdRp, such as the priming loop, were also defined and elaborated on.

### Identification and docking of popular *flavivirus* inhibitors specific to the RdRp region

Various inhibitors of the RdRp region of DENV were selected from literature based upon their compelling inhibitory characteristics (García, Padilla & Castaño, 2017). Subsequently, ten compounds exhibiting potent antiflaviviral activity, were chosen for docking. The 3-D structures of the compounds were downloaded from the PubChem database website saved in SDF format (https://pubchem.ncbi.nlm.nih.gov/;  Kim et al., 2016). The compounds were then docked to the active site of DENV RdRp (as stated above, the active site residues were chosen based on literature) using the Autodock plugin of Chimera (Morris et al., 1998). In each of the docked complexes, the RdRp residues interacting with the compounds were identified and analyzed. The binding affinities were then evaluated, and the inhibitor with the best docked pose was used to build a pharmacophore model. To validate the docked poses of all ten compounds, the docked complexes were superimposed to the GTP-bound crystal structure of Dengue RdRp (PDB code: 2J7W). This validated that all docked poses were within the active site region of the enzyme. The three best docked complexes were then chosen to assess the protein ligand interactions using the Maestro software (Halgren, 2009). This validated the active site residue interactions with the ligands.

### Pharmacophore model generation

Following docking, the complex with the best binding affinity was subjected to a protein-ligand interaction plot using LigandScout (Wolber & Langer, 2005) and Ligplot (Wallace, Laskowski & Thornton, 1995) software. This plot graphically demonstrated the intra-molecular forces that stabilized the compound at the active site of RdRp. Based on these interactions, molecular groups that significantly interacted with the contributing residues were selected to construct the pharmacophoric scaffold. This was accomplished by uploading the compound on the ZincPharmer (Koes & Carlos, 2012) webpage and selecting the pharmacophoric moieties based on the chemical interactions from the interaction plot.

### Results and Discussion

### Assembly of structural and non-structural DENV proteins

**Figure 1:**
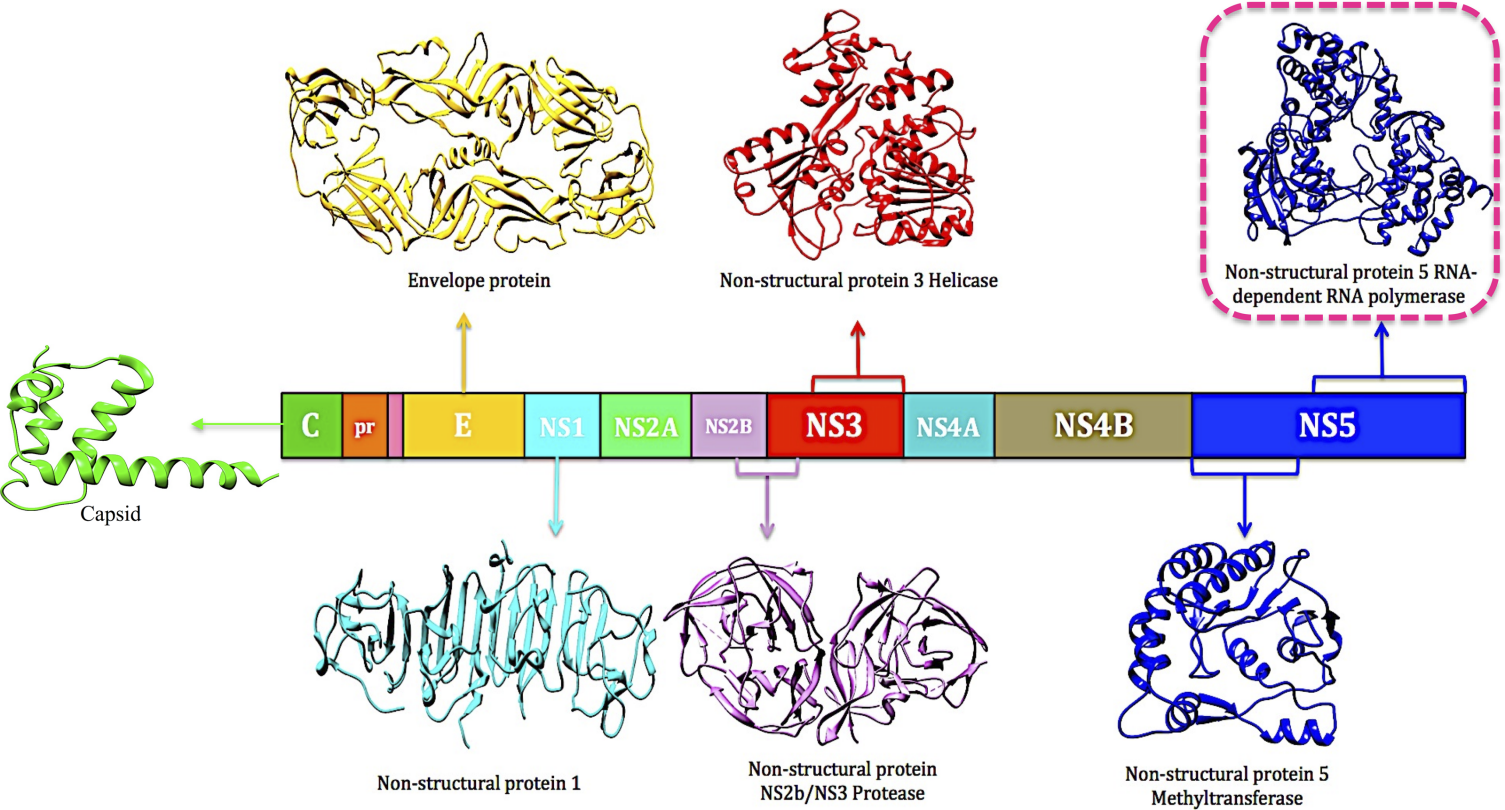
Dengue polyprotein demonstrating available protein crystal structures (PDB codes: 1R6R, 4UTC, 4O6B, 2FOM, 2VBC, 1L9K, 5K5M).

Dengue is an enveloped *flavivirus*, which has a positive-sense RNA genome approximately 11 kb in size (Miller et al., 2008). This RNA encodes three structural proteins that form the components of the virion: the capsid (C), precursor membrane (prM) and the envelope (E). In addition to this assembly, there are seven non-structural (NS) proteins namely NS1, NS2A, NS2B, NS3, NS4A, NS4B, and NS5 (Shu et al., 2004; Perera & Kuhn, 2008; El Sahili & Lescar, 2017). Of the structural and non-structural proteins, crystal structures are available for the capsid (1R6R), envelope (4UTC), NS1 (4O6B), NS2 (2FOM), NS3 (2VBC) and NS5 (1L9K, 5K5M) (Fig. 1).

The NS5 is the largest non-structural protein and comprises of the methyltransferase and RNA-dependent RNA polymerase (RdRp) (Zhao et al., 2015; Klema et al., 2016). The RdRp plays a significant role in RNA synthesis by catalyzing the replication of RNA synthesis via a two-step mechanism, thus validating it as an essential target for antiviral therapy (Ferron et al., 2005; Paschal et al., 2008).

### Sequence and structural analysis of DENV RdRp

**Figure 2:**
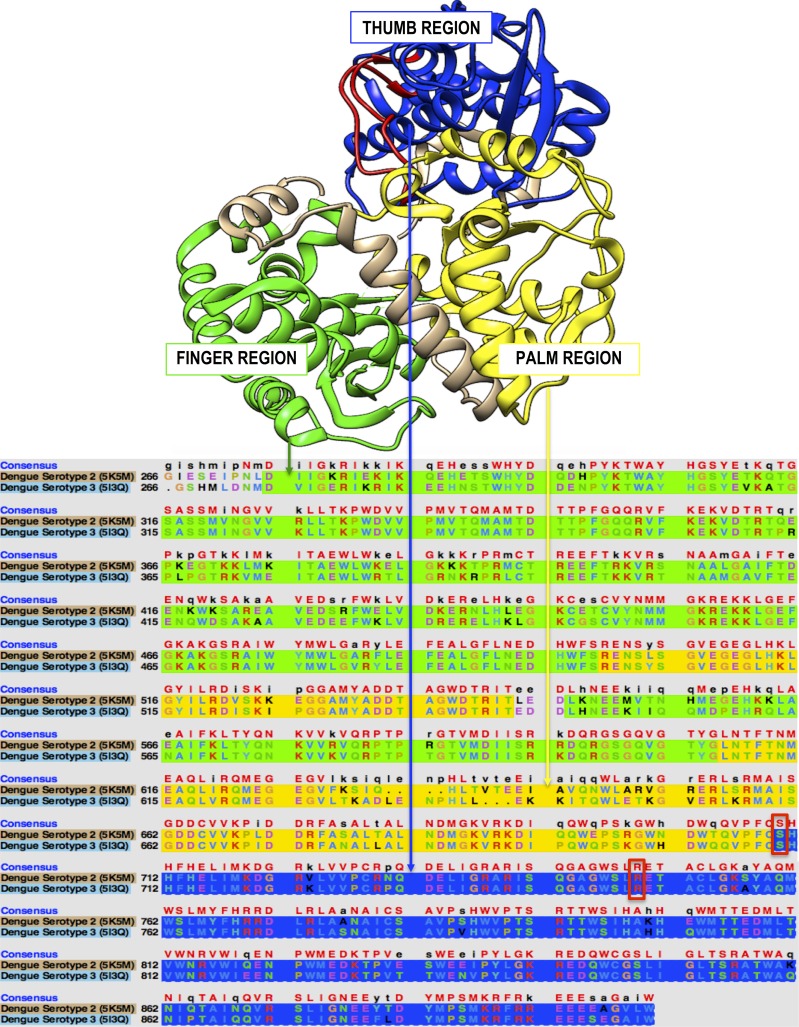
The overall structure and sequence analysis of the RdRp enzyme of DENV serotype 2 and 3. The green colour represents the finger domain (273–315,416–496 and 543–600); the yellow represents the palm domain (497–542 and 601–705) and the blue represents the thumb domain (706–900). The priming loop found in the thumb domain is represented by the red colour (782–809).

The RdRp of DENV is located on the C terminus of the NS5 protein from residue number 266-900 (Fig. 2) (Perera & Kuhn, 2008; Klema et al., 2016).

The highlighted regions of the sequence represent the three domains of the RdRp region (Lu & Gong, 2017). The green highlight represents the finger region, the yellow represents the palm and the blue represents the thumb (Yap et al., 2007; Klema et al., 2016). A general resemblance of amino acids was noted between DENV serotypes 2 and 3 (King et al., 2007; Wu, Liu & Gong, 2015). Regions of maximum resemblance lie between 401 and 441 within the palm region. However, significant variations are prominent within the finger region. This is due to genetic alterations that have caused the DENV virus to mutate (Holmes & Burch, 2000; Holmes & Twiddy, 2003; Hellenthal & Stephens, 2006). This genetic variation is caused by error-prone RdRp, which lacks proofreading activity and generates approximately one mutation per round of genome replication (Elena & Sanjuán, 2005; Sessions et al., 2015). Genetic recombination is also known to cause intra-serotype genetic variation in DENV (Uzcategui et al., 2001; Craig et al., 2003; Holmes & Twiddy, 2003; Perez-Ramirez et al., 2009).

The architecture of the DENV RdRp adopts a canonical right-hand conformation comprising of a finger, palm and thumb domain surrounding its active site (Lu & Gong, 2017). This applies to most polymerases (Ago et al., 1999; Duan et al., 2017). Dengue, however, has a nuclear localized structure (NLS) that plays a major role in its structural formation (Yap et al., 2007; Zhao et al., 2015). The NLS signatures are distributed between the finger and thumb domains from residues 316–415. This region forms the hotspot for interactions with other viral and host proteins (Johansson et al., 2001; Zou et al., 2011; Brooks et al., 2002). Alterations within the NLS region lead to structural destabilization (Pryor et al., 2007; Yap et al., 2007; Malet et al., 2007).

### Finger domain

The finger domain is divided into two subdomains. The first strand is a beta-rich-strand (*β*) subdomain and the fingers found in this strand are termed beta-fingers (Ago et al., 1999). The other strand is rich in alpha-helices (*α*) and therefore the fingers found in this region are alpha-fingers. In addition, the finger region has four flexible loops, *β*1-*α*2, *α*3-*α*4, *α*6-*α*7 and *α*7-*α*8. Overall, the residues in the finger region are from 273 to 600 (Egloff et al., 2002; Galiano et al., 2016; Duan et al., 2017). The finger domain is located at the top of the RdRp enzyme and appears to be more mobile than the other two domains (Poch et al., 1989; Ng, Arnold & Cameron, 2008; Zou et al., 2011).

### Palm domain

The palm domain is a catalytic domain that encompasses a highly conserved folding motif (Malet et al., 2007; Yap et al., 2007). The palm consists of two antiparallel *β*-strands, *β*4 and *β*5, and is surrounded by eight helices which are *α*11-*α*13 and *α*16-*α*20. Of the six conserved sequence motifs located in the palm region, two residues, Ser-710 and Arg-729, are specifically involved in nucleotide triphosphate (NTP) binding and catalysis (Bruenn, 1991; D’Haeseleer, 2006; Ferrer-Orta et al., 2006; Doolittle & Gomez, 2011; Asnet Mary, Paramasivan & Shenbagarathai, 2015; Galiano et al., 2016; Duan et al., 2017).

### Thumb domain

The thumb domain stabilizes the C-terminal end of the RdRp, (Midgley et al., 2012) and is composed of residues 706–900 on the *β*6-*α*23 strands (Yap et al., 2007; Galiano et al., 2016). Of the known polymerase structures, the DENV thumb region shows the most unique structural variation (Paschal et al., 2008; Cramer & Arnold, 2009). The thumb domain contains a conserved sequence motif that forms an antiparallel *β*-sheet wedged between the palm domain and several *α*-helices of the thumb domain (Ng, Arnold & Cameron, 2008; Pierson & Diamond, 2012). This unique structure contributes to the shaping of the RNA template tunnel (Benarroch et al., 2004; Yap et al., 2007; Welsch et al., 2009).

### Priming loop

**Figure 3:**
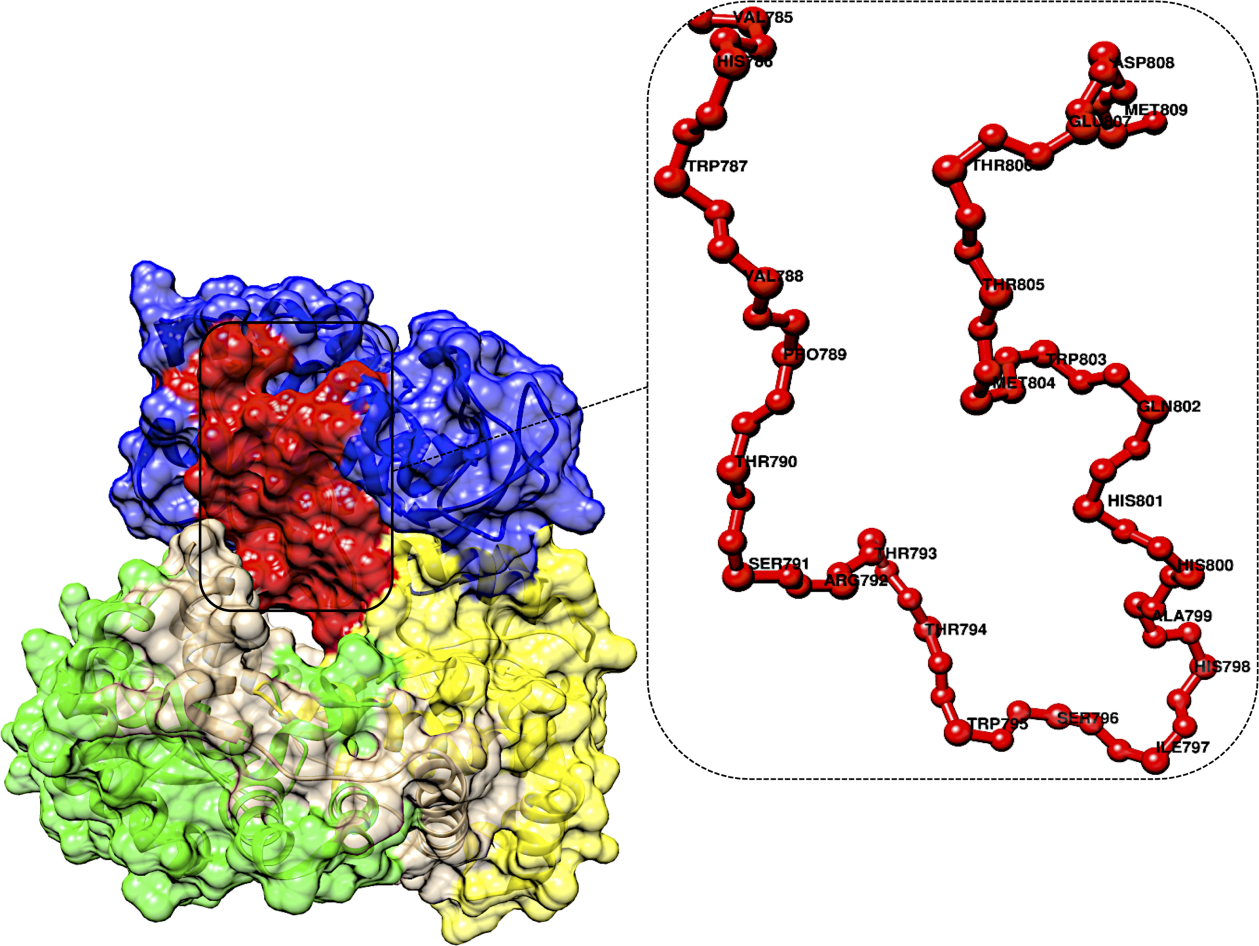
DENV RdRp Priming loop. A closer look into the priming loop region highlighted in red in the DENV RdRp region.

A second loop consisting of amino acids 782–809 forms the priming loop, which partially blocks the active site (Fig. 3). The priming loop plays a key role in initiating the enzymatic activity of the RdRp (Gebhard, Filomatori & Gamarnik, 2011; Selisko et al., 2012; Te Velthuis et al., 2016). Internal interactions, including hydrogen bonds, act to stabilize the priming loop, thus maintaining the orientation of the protein structure (Ng, Arnold & Cameron, 2008; Campagnola et al., 2015). The priming loop is also known as the G-loop because it corresponds to motif G in primer-dependant RdRps. The characteristic “hairpin” structure of the loop is partially disordered in *flavivirus* RdRp structures, suggesting conformational flexibility (Malet et al., 2008; Source et al., 2013).

### Comparative mapping of DENV RdRp active site

**Figure 4:**
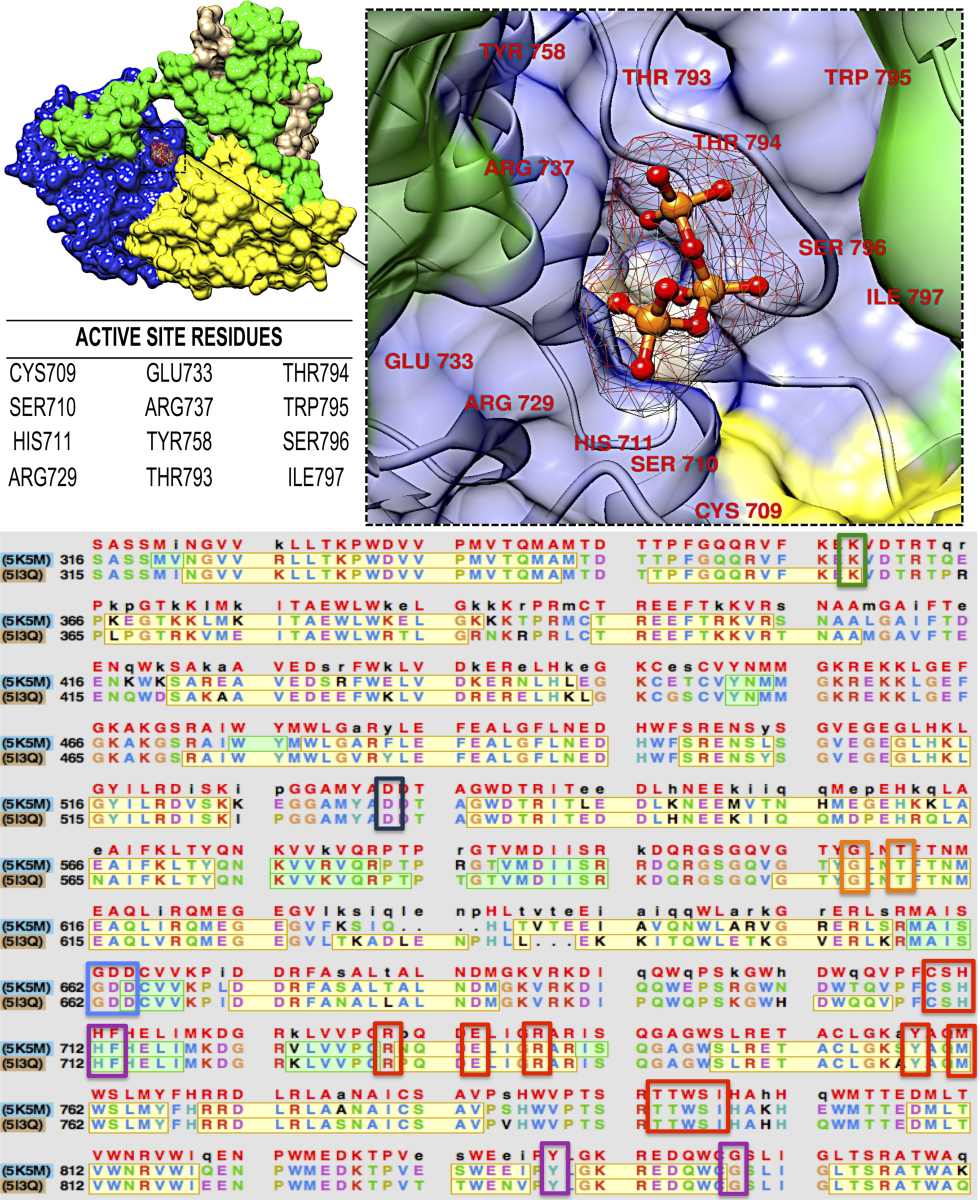
The active site region and sequence analysis of DENV. Active site residues are highlighted in red, Motif A in black, Motif B in orange, Motif C in blue, motif D in green and Motif E in purple.

The RdRp active site is characterized by a conserved region comprising of a glycine-aspartate core section located in the palm domain (Jablonski, Luo & Morrow, 1991; Routhier & Bruenn, 1998; Wu, Liu & Gong, 2015). The active site of DENV is made up of hydrophobic residues Ile797 and Trp795, as well as polar residues Cys709, Ser710, Ser796, Tyr758, Thr793 and Thr794. These amino acids, together with basic residues His711, Arg729 and Arg737, as well as acidic residue Glu733 (Source et al., 2013; Klema et al., 2016), are all located within the palm domain (Ago et al., 1999; Lesburg et al., 1999) (Fig. 4). One of the unique characteristics of the active site is its location between the intersections of two tunnels. The finger and thumb domain form the first tunnel, which is responsible for coordinating the single-stranded RNA, while the second tunnel coordinates the nascent double-stranded RNA (Yap et al., 2007).

Mutations on the active site have contributed to challenges in finding inhibitors for DENV (Mateo, Nagamine & Kirkegaard, 2015), thus analysis of the active site will enable researchers to find broad spectrum inhibitors against both serotypes of DENV.

Seven catalytic motifs, A–G, have been identified for DENV RdRp. These motifs contribute to the sequence and structural conservation of the RdRp active site. Motifs A (Asp533) and C (Asp663, Asp664) contain aspartic acid residues that are universally conserved amongst Flaviviruses. Motif B has a highly conserved RdRp-specific serine-glycine sequence (Gly608, Ser611), which is replaced by threonine in drug-resistant strains (Perera & Kuhn, 2008; Klema et al., 2016; Yap et al., 2007). The glycine adjacent to motif B provides the backbone flexibility needed for conformational switches around the adjacent serine. The sequence is also vital for allowing large-scale conformational changes of the motif B loop. Motif D does not have conserved residues, however, it contains a lysine residue that has been shown to contribute in catalysis. Motif E and G do not contain conserved residues, but contribute to the composition of zinc-binding at RdRp (Yap et al., 2007; Lim et al., 2016; Zhao et al., 2015).

### Conquering targeted therapy with popular drugs

Studies have identified multiple general *flavivirus* RdRp inhibitors, however, there are currently no FDA approved drugs that are specific to all serotypes of the RdRp region of DENV. The development of an antiviral therapy for DENV is further complicated by the fact that protection against one serotype leads to increased vulnerability against the other serotypes (Heinz & Stiasny, 2012). This study therefore seeks to fill the gap between the increase in DENV case reports and absence of antivirals. Over the years, inhibitors that have shown potential as antivirals have come with multiple challenges including elevated toxicity levels. Scientists are therefore still battling to find an inhibitor that is potent, efficacious and non-toxic for the treatment of DENV (Galiano et al., 2016; García, Padilla & Castaño, 2017; Ramharack & Soliman, 2017).

**Figure 5:**
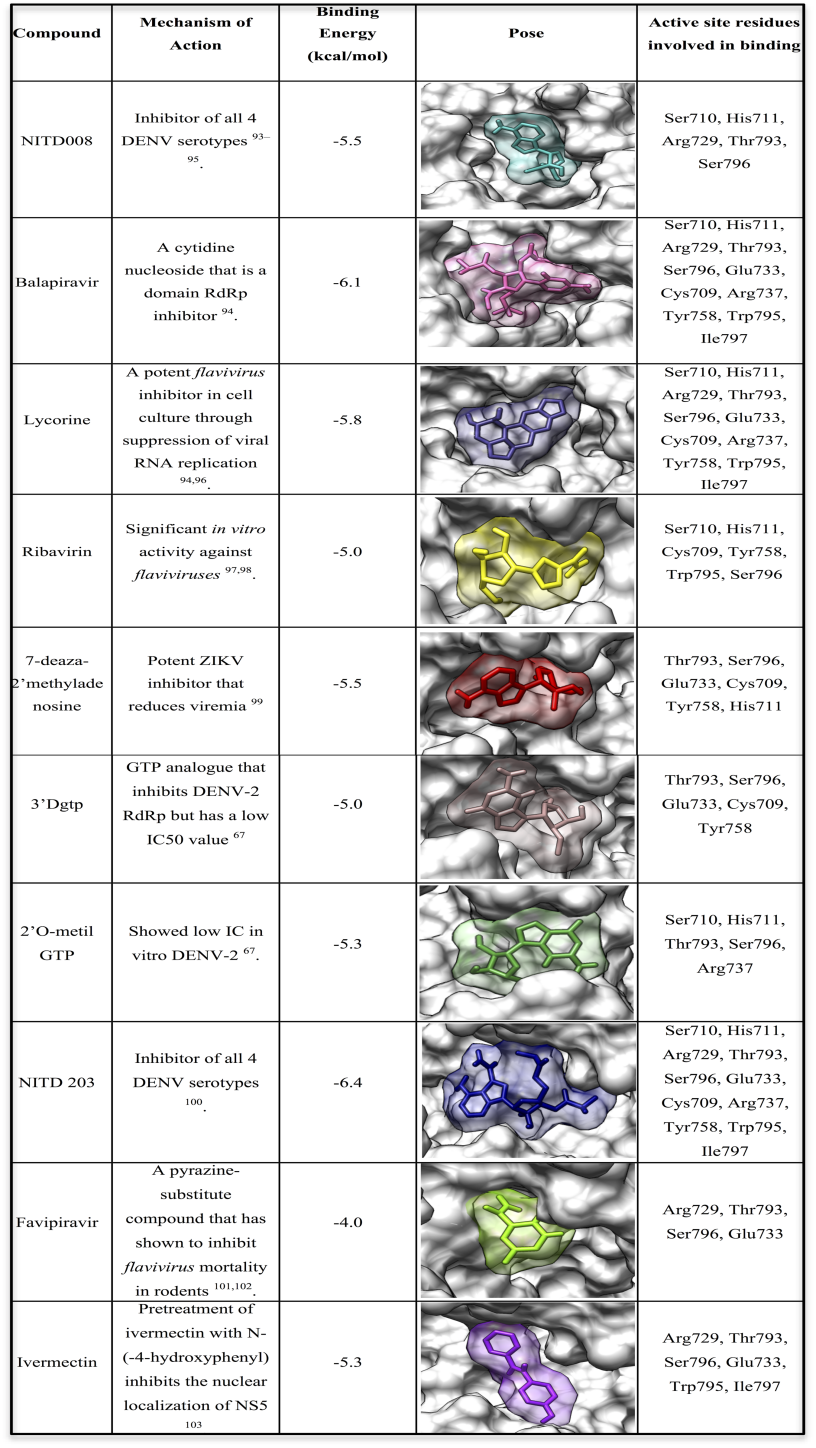
The 10 best docked poses of inhibitors bound to DENV RdRp.

In this study, various potent inhibitors of the RdRp region of DENV were assessed. Based on a study by (García, Padilla & Castaño, 2017), experimental compounds that demonstrated compelling inhibition of DENV were chosen and docked into the active site of DENV RdRp. The ten best docked poses are reported in Fig. 5. The RdRp residues interacting with the docked compounds were identified, thus adding to the requirements needed when designing a possible inhibitor of DENV. Of these residues, Ser796 was found in all ten of the complexes, indicating its importance as an interacting residue for both serotypes.

Various studies have proved NITD008 to be a potent *flavivirus* inhibitor (Yin et al., 2009; Shan et al., 2013; Deng et al., 2014; Deng et al., 2016). The binding affinities, however, showed that NITD-203 had the best docking score (−6.4 kcal/mol). The NITD-203 compound is an adenosine analog that has been shown to have potent competitive inhibition of adenosine triphosphate (ATP) at the active site of RdRp. A study by Chen et al. (2010) identified NITD-203 to demonstrate potent *in vivo* efficacy in a DENV viremia mouse model. It was, however, important to note that the compound did not reach a “no-observable adverse-effect” level. Further studies, thereafter, confirmed that one of the most common adverse effects of nucleoside compounds, such as NITD-203, is mitochondrial toxicity (García, Padilla & Castaño, 2017). This adverse effect dismissed the compound’s progression to FDA approval. Nonetheless, NITD-203 may still be utilized in the development of DENV antiviral therapy through drug optimization.

The intermolecular interactions between a drug molecule and the amino acids in an active site alter its structure and conformation. This allows the drug to stabilize within the docking site. When the binding affinity of a ligand is higher at an active pocket, it is an indication that the ligand is more stable. It was noted from the docking results that NITD-203 showed the greatest stability as seen by the intermolecular forces.

Based on the “prodrug” characteristics of NITD-203, it was chosen as a model to identify specific pharmacophoric elements that are required when designing an efficient inhibitor of all four serotypes. Pharmacophore modeling is a pivotal tool exploited in rational drug design, providing crucial insights into the nature of the interactions between a drug target and ligand. It involves the concept of “privileged structures”, which are molecular frameworks capable of providing useful ligands for more than one type of protein. Pharmacophore models are vital in drug design as they act as templates for screening compounds that have similar structural and chemical features. These ligands could then be used as lead compounds against various diseases (Wolber & Langer, 2005;  Qing et al., 2014).

**Figure 6:**
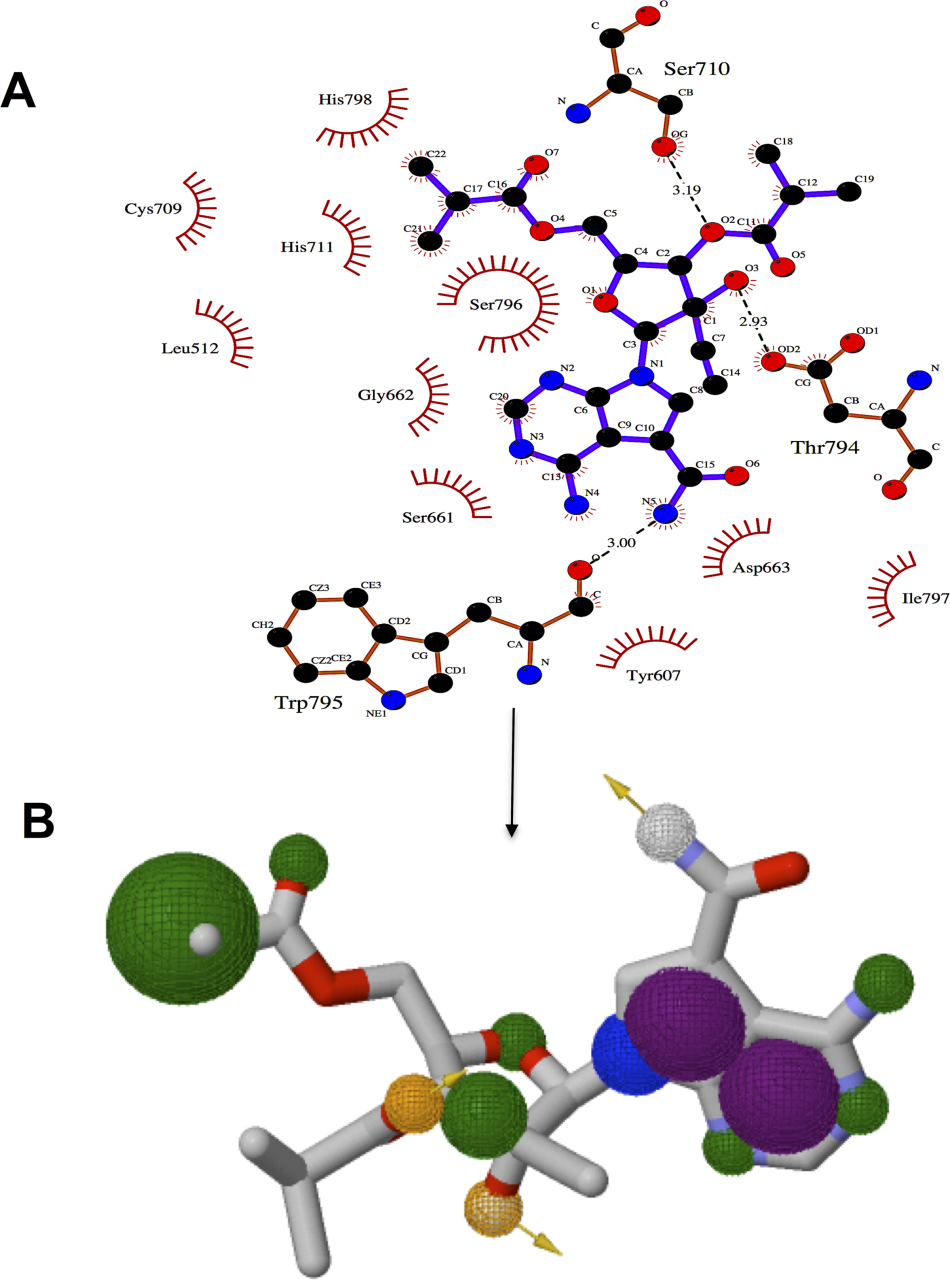
Significant pharmacophoric elements of NITD-203 for the design of target-specific inhibitors of DENV RdRp (A) shows intermolecular interactions of active site residues with NITD-203 (B) shows pharmacophore model, hydrophobic regions are depicted in green, hydrogen donor/acceptor in yellow and aromatic rings in purple.

In this study, we have therefore utilized this pharmacophoric approach to design a model based on NITD-203 that may be used as a stepping stone toward efficient DENV inhibitors. The Ligplus software (Wallace, Laskowski & Thornton, 1995) was used to demonstrate the vital pharmacophoric elements required when designing a DENV RdRp inhibitor. These chemical features were based on active site residue interactions with functional groups of NITD-203 (Fig. 6).

Based on the pharmacophore model identified in Fig. 6, chemical features such as hydrogen bond donors/acceptors as well as aromatic rings are crucial elements that are required in constructing an efficacious DENV inhibitor.

From a structure–activity relationship viewpoint, the second oxygen, third hydroxyl and fifth amino groups are vital within the molecule as they partake in hydrogen bong interactions with Ser710, Thr794 and Trp795 of the active site region. The ligand also formed hydrophobic interactions with amino acid residues Cys709, His711, Leu512, Gly662, Ser611, Ser796, Tyr607, Asp663, Ile797 and His798. These interactions were noted at the pyrrole and pyrimidinyl aromatic rings as well as the methylproponate groups of the ligand.

In summary, we believe that this 3-D structure based pharmacophore model may be used to screen large libraries of compounds to identify potential lead molecules that are target-specific inhibitors against DENV RdRp.

## Conclusion

Dengue is an established *flavivirus* that is causing distress in the lives of many. The development of an antiviral against DENV is further complicated by its manifestation into various serotypes. This augments the need for innovative research methods in DENV drug design. The bioinformatics techniques discussed in this paper will aid in the identification of potential RdRp inhibitors, thus mitigating the effects of DENV in the lives of compromised individuals, as well as prevent the transmission of DENV on a global scale.

### Future perspectives

Using bioinformatic software, the sequence and structure of two serotypes of DENV RdRp were analyzed and a 3-D pharmacophore model was generated based on the active region amino acid residues. Further studies based on these results include:

1. Virtual screening of the pharmacophore model, through chemical databases, to identify potential lead compounds based on docking score and druglikeness. These compounds may then be subjected to molecular dynamic simulations to verify its stability in the RdRp active site. Favourable compounds may then undergo *in vitro* studies for efficacy and toxicity profiling.
2. Drug optimization may also be another avenue in identifying potential inhibiitors for DENV therapy. One of these molecular modification strategies is bioisosteric replacement. This method describes replacing certain molecular groups of a ligand with bioisosteric functional groups that possess similar physiochemical properties of similar biological effects. This may curb the adverse effects that may be caused due to redundant molecular groups. The modified compounds may then be assessed based on their toxicity and efficacy profiles.

##  Supplemental Information

10.7717/peerj.5068/supp-1 Supplemental Information 1 Supplementary data: links for structures of protein and compounds

## Additional Information and Declarations

### Data Availability

The following information was supplied regarding data availability: The links to the third-party data used in this work are provided in the Supplemental File.
