# Supplementary material for: Using bioinformatics tools for the discovery of Dengue RNA-dependent RNA polymerase inhibitors
Source: PeerJ. 2018 Sep 25;6:e5068. doi: 10.7717/peerj.5068 (PMC6161702; doi:10.7717/peerj.5068)
Supplement: Supplemental Information 1 [file peerj-06-5068-s001.docx]

**Using Bioinformatics Tools For The Discovery of Dengue RNA-dependent RNA Polymerase Inhibitors**

Nomagugu B. Ncube^a^, Pritika Ramharack^a^, Mahmoud E. S. Soliman^a*^

**Supplementary Material**

Links for Protein crystal structures:

- <https://www.rcsb.org/structure/5K5M>
- <https://www.rcsb.org/structure/5I3Q>

Links for PubChem structures:

- NITD008: <https://pubchem.ncbi.nlm.nih.gov/compound/44633776>
- Balapiravir: <https://pubchem.ncbi.nlm.nih.gov/compound/11691726>
- Lycorine: <https://pubchem.ncbi.nlm.nih.gov/compound/11822288#section=Top>
- Ribavirin: <https://pubchem.ncbi.nlm.nih.gov/compound/37542>
- 7-Deaza-2’mathyladenosine: <https://pubchem.ncbi.nlm.nih.gov/compound/127927>
- 3’-dGTP: <https://pubchem.ncbi.nlm.nih.gov/compound/148770>
- 2’O-metil GTP: <https://pubchem.ncbi.nlm.nih.gov/compound/2_-O-Methylguanosine>
- NITD-203: <https://pubchem.ncbi.nlm.nih.gov/compound/44633774>
- Favipiravir: <https://pubchem.ncbi.nlm.nih.gov/compound/492405>
- Ivermectin: <https://pubchem.ncbi.nlm.nih.gov/compound/11957587>


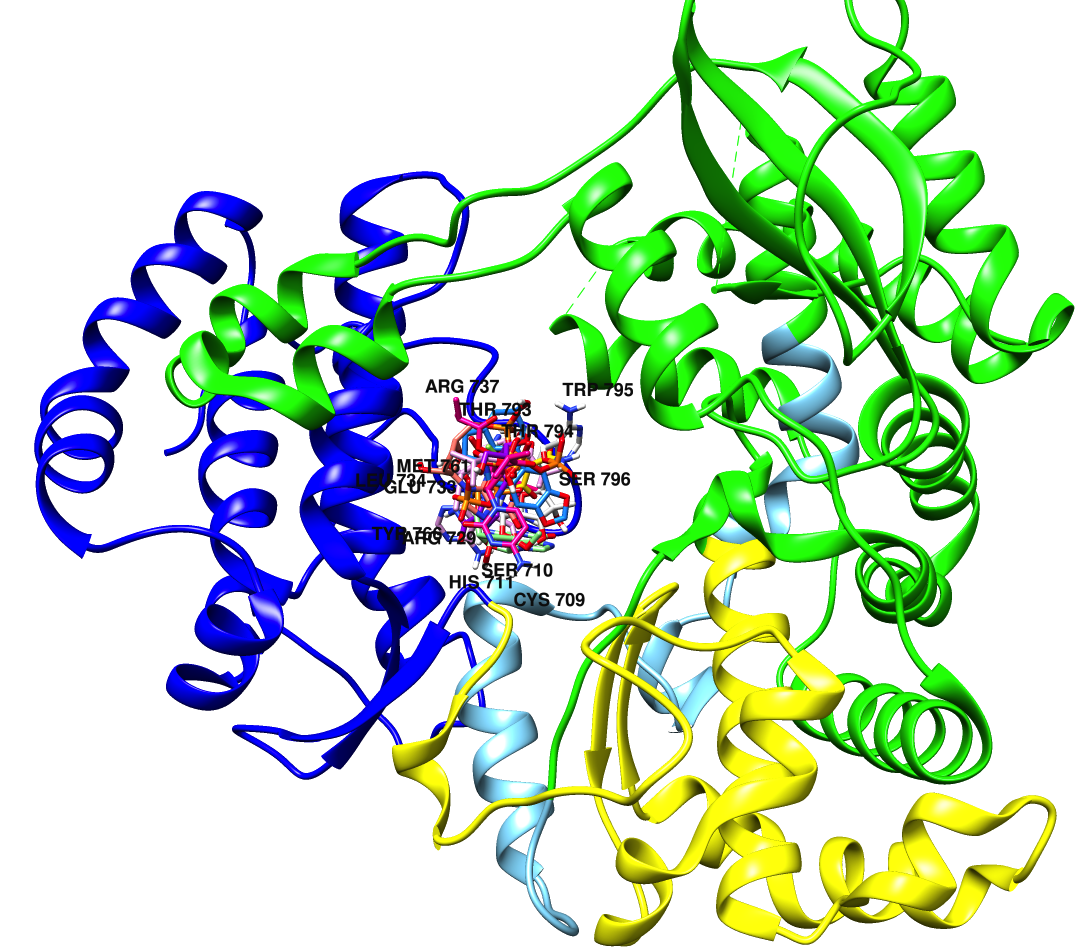


**Figure S1**: Superimposed docked complexes with GTP-bound RdRp (PDB code: 2J7W). Validation of docking to the active site of the enzyme at the region of natural substrate binding.


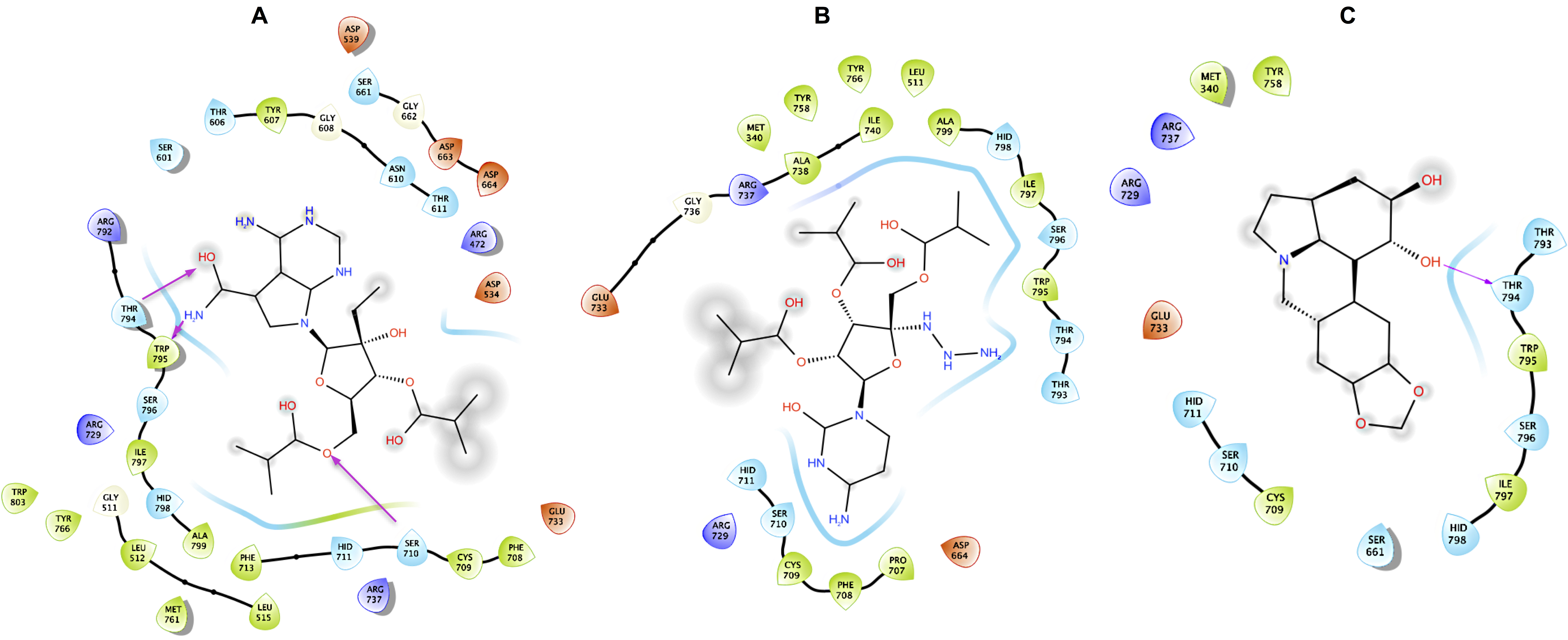


Figure S2: Protein-ligand interaction plots (A) NITD-203 RdRp complex, (B) Balapiravir RdRp Complex, (C) Lycorine RdRp complex.
